# Effectiveness of a peer group-based online intervention program in empowering families of children with disabilities at home

**DOI:** 10.3389/fped.2022.929146

**Published:** 2022-10-24

**Authors:** Rie Wakimizu, Akemi Matsuzawa, Hiroshi Fujioka, Kaori Nishigaki, Iori Sato, Seigo Suzuki, Naoko Iwata

**Affiliations:** ^1^ Department of Child Health and Development Nursing, Division of Health Innovation and Nursing, Faculty of Medicine, University of Tsukuba, Tsukuba-city, Japan; ^2^ Department of Comprehensive Development Nursing, Graduate School of Health Sciences and Faculty of Medicine, Hokkaido University, Sapporo, Japan; ^3^ Department of Nursing, Faculty of Health Sciences, Ibaraki Prefectural University of Health Sciences, Ibaraki, Japan; ^4^ Department of Child Health Nursing, Graduate School of Nursing Sciences, St. Luke’s International University, Tokyo, Japan; ^5^ Department of Family Nursing, School of Health Sciences and Nursing, Graduate School of Medicine, The University of Tokyo, Tokyo, Japan; ^6^ Department of Pediatric Nursing, Faculty of Medicine, Tokyo Medical University, Tokyo, Japan; ^7^ Tsukuba University Hospital, Medical Liaison and Patient Support Services Center, Ibaraki, Japan

**Keywords:** children with disabilities, caregiver, family empowerment, well-being, quality of life, online intervention program, effectiveness verification study

## Abstract

**Background:**

The empowerment of families raising children with disabilities (CWD) is crucial in maintaining their health. We developed an evidence-based, family empowerment intervention program focusing on social resource utilization and reducing care burden.

**Objective:**

This study aimed to determine the program's effectiveness in promoting family empowerment.

**Methods:**

We compared an intervention group that started the online intervention program a week after initial evaluation and a group that received delayed intervention (waitlist-controlled group) at three time points: initial (T1), post-course (T2), and follow-up (T3). The required sample size was 52.

**Results:**

There were 60 participants who applied to the program. One participant dropped out due to scheduling issues, and the others were assigned to either the intervention group (*n* = 29) or the waitlist-controlled group (*n* = 30). Those who responded to the baseline questionnaire (T1: 26 from the intervention group; 29 from the waitlist-controlled group) comprised the final sample. Among them, 20 members of the intervention group and 20 of the waitlist-controlled group attended all four sessions (completion rates of 77% and 69%, respectively). The attendance rate for sessions 1–4 was 94%, 89%, 81%, and 83%, respectively. The participant numbers in each session ranged from 5 to 18 per month. The baseline outcome score did not differ between the groups. The primary outcome, family empowerment, measured using the family empowerment scale (FES), was significantly higher at T2 for the intervention group than in the waitlist-controlled group and was sustained in the sensitivity analysis. The intervention group's FES, in the family relationships (FA) and relationships with service systems (SS) subdomains, increased significantly, unlike involvement with the community (SP). The intervention group experienced lower care burden and higher self-compassion, especially in the isolation and over-identification items of the self-compassion scale-short form (SCS-SF). The intervention group's FES (total, FA, SS) and SCS-SF (total, common humanity, isolation) changed significantly between T1 and T2, and all, except common humanity, were sustained up to T3; this group's FES (SP) and SCS (negative score, over-identification) changed significantly between T1 and T3. The waitlist-controlled group's FES (total, FA) and SCS (total) changed significantly and were sustained between T2 and T3.

**Conclusions:**

The developed intervention program promotes family empowerment in families of CWD.

**Clinical Trial Registration:**

This study is registered as a clinical trial in the UMIN Clinical Trials Registry (https://center6.umin.ac.jp/cgi-open-bin/ctr/ctr_view.cgi?recptno=R000050422, UMIN000044172).

## Introduction

Raising children with disabilities (CWD) requires more time and skills than raising healthy children (1). Therefore, families of CWD face much greater health-related and daily-life-related risks than families with healthy children. Especially, mothers, the primary caregivers, make long-term time commitments and attend to the care needs of their CWD. However, they often feel burdened by these responsibilities and have higher stress levels, leading to poorer health (2–5). They also experience several limitations in social participation, such as in the workforce (6). Therefore, developing support systems for families raising CWD is crucial.

This cannot be achieved through public services alone, and it is essential to promote the independence of families living and raising their children in the manner they desire. In this context, family empowerment—families' ability to control their lives and promote the collaborative raising of CWD—has garnered considerable attention (7). Koren et al. proposed a conceptual framework of empowerment for families raising CWD in the community [“empowerment of family (internal) relationships,” “empowerment of relationships with service systems,” and “empowerment of interactions with the community”] (8). As noted above, raising CWD requires a great deal of time and skill and has a significant impact on the primary caregiver and other family members. Therefore, these families need to be able to cooperate with the family members, as well as to access a variety of healthcare and social services in the community. Also, those families require to access the several services they need on their own, and collaborate with healthcare professionals, and with the government in the community. When these families are able to collaborate within the family, with healthcare services, and with the community to align their lives, family empowerment contributes to improved positive outcomes for families. In a previous study, family empowerment determines the well-being of mothers (9) and the quality of life (QOL) of their CWD (10).

In Japan, owing to advanced medical care, children with severe disabilities are increasingly receiving care early on. The number of children needing medical care after acute treatment is increasing rapidly, many of whom live with their families (11). These families need empowerment so that they can live and raise their children confidently.

In previous studies, to comprehensively identify the factors in the family empowerment model (FEM), we conducted in-depth interviews with 34 families [mother, father, and siblings (aged ≥ 2)] of children with severe motor and intellectual disabilities living at home (12), and a questionnaire survey with 158 nurses and government officials using the Delphi method (13). Subsequently, we conducted a self-administered questionnaire-based survey on the FEM with 1,659 families raising CWD nationwide to develop and verify the model (14). This study aimed to comprehensively explore factors related to family empowerment among families raising children with severe motor and intellectual disabilities. The results of this study indicated that family empowerment was influenced by these families' perceptions of “utilization of social resources” and “reduce their caregiving burden” to a higher level of family empowerment. We measured not how well families were utilization of social resources, but whether they themselves perceived that they were utilization of social resources. Moreover, the caregiver burden was measured in terms of the subjective sense of caregiving burden that the family members felt in caring for their children. These were, however, observational studies.

In the current study, through implementation research, we verified whether an intervention program employing the previously identified factors—increasing families' awareness of social resource utilization and reducing caregiver burden—could increase family empowerment. Further, we determined which aspects of FEM—“family (internal) (FA),” “service system (SS),” or “community (SP)”—are empowered by the intervention. We developed a family empowerment intervention program by focusing on family empowerment-related factors, mainly “(awareness of) social resource utilization” and “reduction of caregiver burden.” The program's pilot test revealed its efficacy in empowering families raising CWD at home (15). Several intervention programs for those families have been developed and evaluated. In one study, Borek et al. (2018) developed a group-based intervention program for families of CWD to improve health and well-being to change behavior by promoting empowerment and resilience (16). In another study, Bourke-Taylor et al. (2022) developed a workshop to educate and empower mothers of CWD to improve their health behaviors (17). In both studies, the effectiveness of the program's interventions has been scientifically verified. However, there are few feasible and evidence-based programs (18). We conducted the program to establish, through a wider sample, whether program participation improves family empowerment. This is the first program development and interventional study focusing on empowering families of CWD in Japan.

## Materials and methods

### Study design and setting

**Figure 1:**
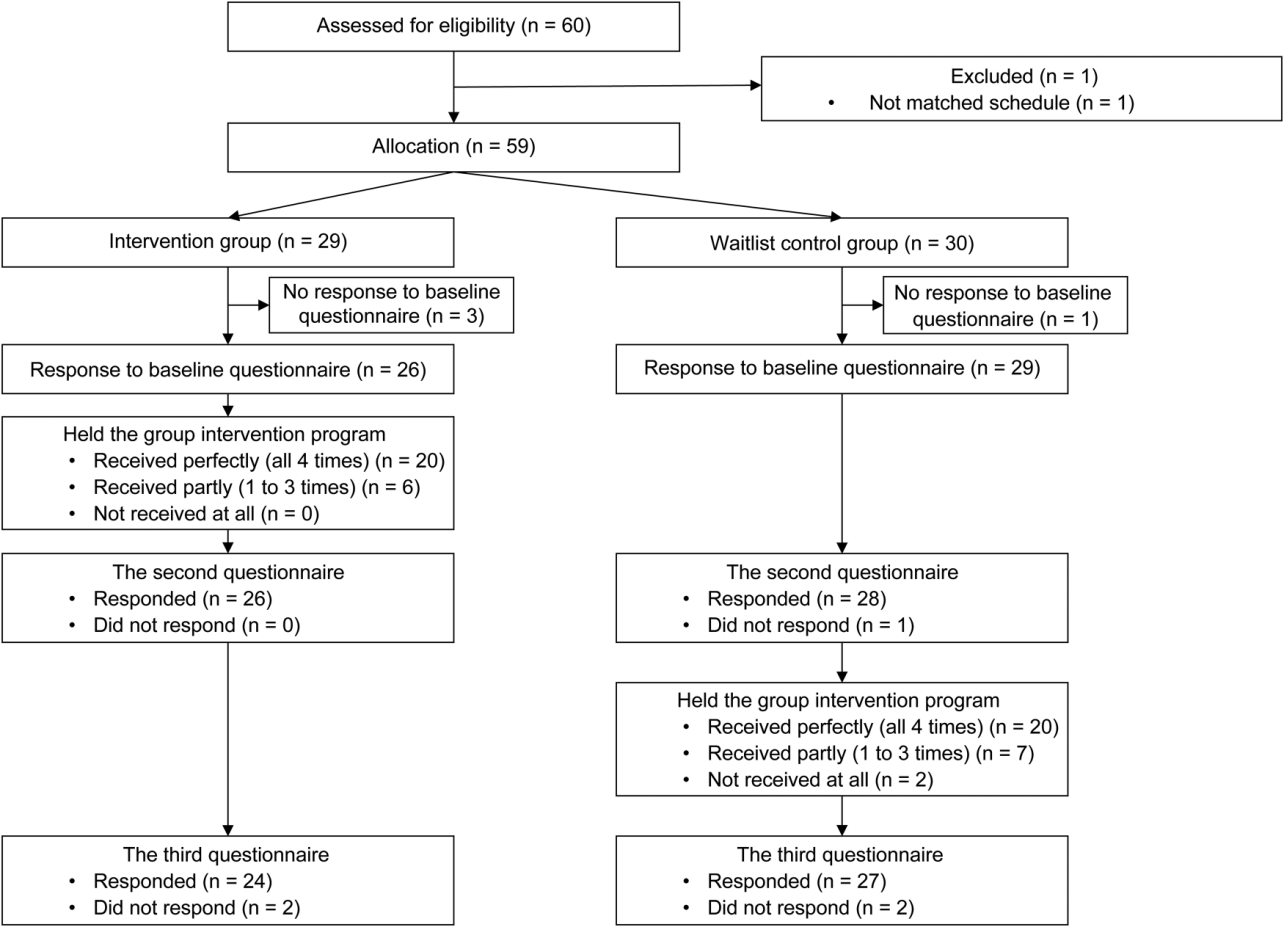
Participant flow chart.

This is a non-randomized, waitlist-controlled trial, comparing the group starting the family empowerment program a week after initial evaluation (intervention/early group) and the group receiving the delayed intervention (waitlist-controlled/delayed group). The program commenced in July 2021 in Japan, with participants being recruited between April and December 2021. This study is registered as a clinical trial in the UMIN Clinical Trials Registry (UMIN000044172). The study protocol was prepared following the Standard Protocol Items: Recommendations for Intervention Trials (SPIRIT) 2013 statement (19). The trial and flow of participants are illustrated in Figure 1. The schedule of enrollment, interventions, and assessments are presented in Supplementary Figure S1.

### Sample size

The required sample size was estimated through *F*-tests (ANCOVA) using G*Power software version 3.1.9.7 (20), based on data from the pretest implemented with the same participant population and program content in a previous study (15). The required sample size was 52, assuming an effect size of 0.33, a test power of 80%, and an *α* value of 0.05.

### Inclusion and exclusion criteria

Participants were family caregivers of children with physical, intellectual, or developmental disabilities living at home. The type of disability was not limited. Participants who could not read or write in Japanese or connect to the Internet were excluded.

### Recruitment and ethics

In total, 20,000 leaflets explaining the study and requesting cooperation were sent *via* post to authorities of organizations catering to CWD, such as specialized medical institutions and rehabilitation centers for children with physical disabilities, university hospitals, specialist pediatric hospitals, medical welfare departments of local governments, and special-needs schools for children with physical impairment or chronic diseases.

Interested authorities displayed the program handouts within their facilities and distributed them among people fitting the inclusion criteria and explained the study to them. These handouts contained the general outline and schedule of the program, its target participants, and the intended implementation method. Interested participants could then access a dedicated website using the handout information to apply to the program, thus consenting to participation. The study was conducted in accordance with the ethical standards of the relevant ethics committee and the Declaration of Helsinki (1975) and its later amendments. This study was approved by the Institutional Review Board of Tsukuba University (Approval No. 1420).

### Program development

Using the intervention mapping (IM) approach, we developed a peer support program for promoting behavioral change and empowerment of families raising CWD (21). As the theoretical basis for program development, we adopted Bandura's social cognitive theory (22) and the taxonomy of behavior change techniques (BCTs) (23), which describe practical intervention methods for behavioral changes. The process (15) and protocol (24) of the intervention development are described in separate papers.

#### Program overview

Four group sessions were held every Saturday afternoon on Zoom®. Participants received their program materials *via* mail about 1 week before beginning the program, to allow advanced checking of the content. All participants gathered online at the beginning of the sessions and during the summary time and were divided into small online rooms (breakout rooms) of two to four groups (three to six participants in each group) for sharing and exchanging ideas at other times. When there were less than five participants, the entire session was conducted in one group (no breakout rooms) to avoid constraints on group dynamics due to limited participants. In the first session, participants were grouped based on similarity in disability type of their CWD. The main program facilitator and researchers decided on the subsequent groupings considering participants' comments and responses, with the objective of allowing interactions among several participants and parents of children of different ages, disabilities, and developmental stages. Here, we aimed to promote the exchange of experiences and opinions from different perspectives and provide self-monitoring opportunities.

#### Program content

In the first session, each participant created and shared an eco-map (25) describing their lives, disabled children, and families. Furthermore, participants identified the three levels of family empowerment in their personal and their family's life: what they can do within the family (FA), with service providers (SS), and in the community (SP). Here, we aimed to promote active group dynamics, which required their realizing each other's situations, obtaining new suggestions by objectively reviewing their personal and family lives, making comparisons with other participants' lives, and gaining a basic understanding of family empowerment (Table 1).

**Table 1:** Purposes and content of the program.

Session	Purpose	Content	
Session 1	Understanding the current situation of children and families	Reflecting on the status of their personal and the child's lives by creating and sharing eco-maps	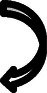 Homework: Create a life chart (weekdays, holidays, and others)
Session 2	Reflecting on the actual lives of the child and family and identifying their desired lives	Sharing their daily lives with other participants on the “Homework: a life chart,” and thinking about their issues and the life they would like to lead	
Session 3	Setting goals for the desired life with the child and family	Sharing goals for the lives they want and setting positive and specific goals	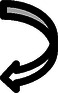 Homework: Practice with the goal of a desired life
Session 4	Reflecting on what they have done to achieve their desired goals for the lives of their child and family	Sharing their actual behaviors and its changes toward their goals, as well as their self-assessments, and recognizing the challenges facing them in their current and desired lives	

In the second session, participants shared the life chart they had created as homework after the first session, identified issues in their lives, and spent time envisioning their personal and their family's desired life. In the third session, participants set clear goals for their personal and their family's desired life, planned and verbalized specific action goals for each level, FA, SS, and SP, and exchanged opinions among themselves. In the fourth session, participants shared opinions on what they worked on as homework after the third session and reflected on the entire program (Table 1).

Throughout the program, attention was paid to clear goal setting based on the characteristics of families raising CWD, providing information, understanding the barriers to benefits, informing them of health and life-related risks, encouraging self-monitoring, peer support, self-compassion, and recognition of family empowerment.

#### Program operations

In each session, two groups of four to six researchers were created, who carried out sessions on a rotation basis for 5 months. The main facilitator was fixed for each month, and three to five sub-facilitators promoted work sharing and opinion exchange among participants in the breakout rooms. As video conferencing was used, in each session, one researcher was assigned to assist participants with their online communication status and file sharing.

#### Intervention tools

Three types of tools were prepared for the program: (1) a text workbook, (2) a supplementary reader with illustrations and descriptions of family empowerment elements, and (3) instructions for an online conference system; (1) and (2) were mailed 1 week before the first program, and (3) was made available online. To facilitate participation, the workbook was printed in color for readability and the homework was printed in large font for usability. A certificate of completion was included at the end of the workbook, and to enhance self-compassion, the researcher emailed a PDF certificate to participants after the last session concluded. Additionally, illustrations in the supplementary reader were used as tools to promote awareness of family empowerment. The three levels of family empowerment were explained and illustrated so that participants could easily refer to them during the program and identify family empowerment elements in their situations.

#### Facilitators

All facilitators were professionals involved in pediatric care, educators, or researchers in pediatric or family nursing. Breakout room facilitators were professionals with a comprehensive understanding of CWD and their families or experienced in working with CWD and their families in clinical settings.

Further, to standardize program implementation, a “facilitation book” was created. This facilitation book's contents explained the program in written scenes, the time required for each task, and guidelines for maintaining facilitation quality.

### Data collection

Group intervention programs were organized every month from July to December 2021; each consisting of four group sessions, with one held every week. After applying, participants were assigned to either the intervention or the waitlist-controlled group and informed of the month they would undertake the program. To prevent biases in their responses due to beliefs about the assigned group, participants were only informed of their participating month, and not whether they were in the intervention or the waitlist-controlled group.

The data were collected through online surveys. The contact person sent three emails requesting participants to respond to surveys per the following schedule: For the intervention/early group, the initial assessment (T1) was conducted before the first session, in the preceding week of the program initiation. The post-course assessment (T2) was conducted immediately after the 4-week program (within 1 week). The follow-up assessment (T3) was conducted 1 month after T2. Participants who did not complete any survey were reminded to do so at least once. The schedule was the same for the waitlist-controlled group, for whose members the program commenced 4 weeks after it commenced for the intervention/early group. Thus, the waitlist-controlled group responded to the T1 survey 5 weeks before the program commencement, T2 immediately before the program, and T3 immediately after the program's completion.

Participant responses were directly imputed into an online survey system. The password-protected datasets were only accessible to the research team and backed up every 2 weeks. The correspondence table for assigning participants to the intervention/early and waitlist-controlled groups was maintained by an investigator other than the one conducting the analysis. Participants were assigned IDs linked to their response data, and each participant's T1–T3 responses were linked. In cases of multiple responses (duplicates) from the same person at the same time point, we used the latest one, considering it to be the revised response. After the T3 survey, the data were downloaded from the system and stored as primary raw data.

### Outcomes and measurement methods

The main outcome, family empowerment, was measured at T1–T3 using the Japanese version of the family empowerment scale (FES) (26). This 34-item scale evaluates how families collaborate to exert control over their lives in three aspects: family (internal) relationships (FA), relationships with service systems (SS), and involvement with the community (SP). The scale employs a five-point Likert scale to calculate the total score and three subscale scores. If the participant had responded to at least half of the items, the missing items were imputed with the mean value of those responses. Higher scores indicated higher levels of family empowerment.

The secondary outcomes were caregiver burden, awareness of using social resources, self-compassion, and the QOL of primary caregivers. Caregiver burden was measured at T1–T3 using the short form of the Japanese version of the Zarit Caregiver Burden Interview (J-ZBI-8) (27). The ZBI-8 evaluates care burden based on physical and mental loads and limitations on social activities. The eight items are evaluated on a five-point scale, with the total score indicating the overall caregiver burden. Here as well, if the participant had responded to at least half of the other items, the mean value of those other responses was entered. Higher scores indicate a greater caregiver burden.

Furthermore, two new sleep-related items, found in previous research (28) to assess a major burden on parents of CWD, were added at T1–T3. The first assessed frequency of waking for nighttime care: “How often do you have to wake up to care for a child with a disability at night?” The responses were “every night,” “several nights a week,” “several nights a month,” “never,” and “other (specify).” The second inquired about the mean daily sleep time in half-hour increments.

Based on previous research (14) and discussions among the researchers on social resource utilization, we formulated two questions. (1) “Do you feel you can properly utilize social resources?” This was asked at T1–T3, with responses, “I often utilize social resources,” “I sometimes utilize social resources,” “I do not utilize social resources very often,” or “I never utilize social resources.” (2) “Compared to when you responded to the previous survey, do you feel you can now properly utilize social resources?” This was asked at T2–T3, with responses, “I now never utilize social resources,” “I no longer utilize social resources very often,” “No change,” “I now sometimes utilize social resources,” or “I now often utilize social resources.”

Self-compassion was measured at T1–T3 using the short form of the Japanese version of the self-compassion scale (SCS) (29). The 12-item SCS assesses six domains, with two items each, on a five-point scale: “(i) self-kindness,” “(ii) self-judgment,” “(iii) common humanity,” “(iv) isolation,” “(v) mindfulness,” and “(vi) over-identification,” thereby providing six subscale scores. If the participant had responded to the other question on the same domain but missed answering a single question, the missing item was imputed with the provided response. Further, we get a positive score by combining (i), (iii), and (v) and a negative score from (ii), (iv), and (vi); higher scores indicate greater or less self-compassion, respectively. The total score was a combination of the positive and the reversed negative score, and higher scores indicated greater self-compassion.

Primary caregivers' QOL was measured at T1–T3 using the Short Form-8 (SF-8) (30), an eight-item scale with responses on a five- or six-point scale. Scores were calculated with a proprietary scoring algorithm and based on a distribution with 50 points as the standard value, with higher scores indicating better health-related QOL. Missing values were handled through the scoring algorithm.

Additionally, data on the following participant attributes were collected at T1: relationship with the child, age (in 10-year increments), marital status, cohabitation with partner, highest education level, occupation, annual household income, and cohabitant family members. The child's attributes assessed at T1 were as follows: gender, age, diagnosis, age at diagnosis, school enrollment status, disability status, and care required. At T2 and T3, any changes in the information provided were to be reported in detail. To avoid forcing participants to respond, a “no response” option was provided, allowing them to proceed without responding.

Furthermore, for program evaluation, the surveys were taken immediately after taking the program and 1 month later to assess participants' feelings toward the program. This was to find out whether they had told their family members about its content or learnings, whether they would recommend the program to friends, how the program should be propagated, and recommendations about its dates and length, and additional space was provided to record any other responses.

### Data analysis

The primary outcome was FES total score at T2. The secondary outcomes were improvement in the family empowerment subdomains (FES subscale scores at T2), aspects the program worked on directly [caregiver burden, social resources use (awareness), self-compassion at T2], and the comprehensive effects of the program (health-related QOL at T2). The persistence of the intervention effect at T2 (each outcome at T3 in the intervention/early group) and its reproducibility observed in the intervention/early group (each outcome at T3 in the waitlist-controlled group) were also validated.

Responses with which outcome scores could be calculated were considered valid responses. Data from participants deviating from study protocols, such as being absent for sessions, were used in the originally allocated groups for intention-to-treat (ITT) analysis. First, descriptive statistics were calculated for participants and their children's attributes, with outcome scores at T1. Mean and standard deviation were calculated for interval scales, and frequency and proportion were calculated for ordinal and nominal scales. The groups were compared using Welch's *t*-test, Mann–Whitney's *U* test, or Fisher's exact test. The attributes and outcome scores at T1 were compared only for participants with valid responses for the FES total score at T2.

In the primary analysis, we used the FES total score at T1 as the covariate and compared the groups using analysis for covariance (ANCOVA), with the FES total score at T2 as the dependent variable. As a premise for ANCOVA, we confirmed there was no significant interaction between the independent variable (intervention/early or waitlist-controlled group) and the covariate (FES total score at T1). In case of a significant interaction, the groups were compared using ANOVA without considering the score at T1. The level of statistical significance was set at 5%. Subgroup analyses were performed on children with severe motor and intellectual disabilities, children depending on medical care, and single parents. The point estimate and 95% confidence interval of the intervention effect in each subgroup were compared to zero and the clinically meaningful change (CMC) score. Furthermore, we examined whether the increase in the FES total score from T1 to T2 was clinically significant. The CMC for FES was not computed. Based on a review (31) suggesting the minimum clinically important difference, we used 0.5 SD of the CMC, and based on a previous study (14), considered the SD to be 16.9; we set the CMC as 8.45.

For secondary results, the outcomes at T2 were compared in the same way through ANCOVA or ANOVA. Outcomes from ordinal scales were compared using the Mann–Whitney *U* test. A paired *t*-test was used to examine the sustainability of the intervention effect at T2, by comparing T1 and T3 scores of the intervention/early group. A paired *t*-test was used to assess the intervention effect's reproducibility in the intervention/early group in the waitlist-controlled group by comparing the T2 and T3 scores of the latter.

We performed a sensitivity reanalysis on our primary analysis in which the change in the FES total score for those who dropped out between T1 and T2 was zero. A per-protocol analysis was performed on participants who attended all four sessions (group) and the other participants (group). Attributes with differences between the groups at T1 were treated as covariates in ANCOVA, and subgroup analyses were also performed.

## Results

### Program summary and participants

Initially, 60 participants applied to the program. One participant dropped out due to scheduling issues, and the others were assigned to either the intervention group (*n* = 29) or the waitlist-controlled group (*n* = 30) (Figure 1). Those who responded to the baseline questionnaire (T1: 26 from the intervention group; 29 from the waitlist-controlled group) comprised the final sample. Among them, 20 members of the intervention group and 20 of the waitlist-controlled group attended all four sessions (completion rates of 77% and 69%, respectively). The attendance rate for sessions 1–4 was 94%, 89%, 81%, and 83%, respectively. The participant numbers in each session ranged from 5 to 18 per month.

We did not compare background attributes and outcome scores at T1 between respondents and non-respondents, as only a few participants dropped out of the study. Imputation of missing values was not performed for the analysis. Almost all participants were mothers, in their 40s, had higher education, and lived with a spouse or partner. Their employment status and incomes varied, but there were no other significant differences between the groups (Table 2). Concerning family structure, the majority of CWD in the waitlist-controlled group had siblings.

**Table 2:** Participant characteristics.

	Intervention group (*n* = 26)		Waitlist-controlled group (*n* = 29)						
	*n*	%	*n*	%	Effect size (Cohen's *h*)	*P* (Fisher exact test)	*P* (Mann–Whitney test)		
Baseline characteristics of participants (parents)									
Relationship with the child									
Mother	26	100.0	28	96.6	0.37	1.000	–		
Father	0	0.0	1	3.4	−0.37				
Age (years)									
20–29	1	3.8	0	0.0	0.39	0.812	0.787		
30–39	2	7.7	3	10.3	−0.09				
40–49	18	69.2	22	75.9	−0.15				
50–59	5	19.2	4	13.8	0.15				
Marital status									
Married	24	92.3	24	82.8	0.29	0.672	-–		
Widowed	0	0.0	1	3.4	−0.37				
Divorced	2	7.7	4	13.8	−0.20				
Cohabitation status with partner									
Cohabit at home	22	84.6	23	82.1	0.07	1.000	–		
Working away from Home	1	3.8	1	3.6	0.01				
Do not cohabit at all	3	11.5	4	14.3	−0.08				
Highest level of education									
Junior high school	0	0.0	1	3.4	−0.37	0.483	0.145		
Senior high school	2	7.7	5	17.2	−0.29				
College/vocational school	7	26.9	9	31.0	−0.09				
University/graduate school	17	65.4	14	48.3	0.35				
Occupation									
Full-time employee	5	19.2	4	13.8	0.15	0.349	–		
Self-employed	1	3.8	4	13.8	−0.37				
Part-time employee	9	34.6	11	37.9	−0.07				
Other worker	1	3.8	4	13.8	−0.37				
Full-time homemaker	10	38.5	6	20.7	0.39				
Household income (billion Yen/year)									
<3	2	8.0	2	7.7	0.01	0.799	0.712		
3–5	6	24.0	8	30.8	−0.15				
5–7	5	20.0	3	11.5	0.23				
7 –10	6	24.0	9	34.6	−0.23				
≥10	6	24.0	4	15.4	0.22				
Living with adults other than parents (e.g., grandparents)									
Yes	11	42.3	10	35.7	0.14	0.781	–		
No	15	57.7	18	64.3	−0.14				
Living with child(ren) other than the child with disability (e.g., sibling(s))									
Yes	13	50.0	22	78.6	−0.61	0.045	–		
No	13	50.0	6	21.4	0.61				
Living with multiple children requiring special healthcare									
Yes	4	15.4	9	34.6	−0.45	0.199	–		
No	22	84.6	17	65.4	0.45				
Baseline characteristics of participants’ children with disability									
Gender									
Female	8	30.8	8	29.6	0.02	1.000	–		
Male	18	69.2	19	70.4	−0.02				
School attendance status (only among school-age children)									
Visit to school	18	100.0	25	100.0	0.00	1.000	–		
Invite teacher to home	0	0.0	0	0.0	0.00				
Condition of motor function: Posture without someone's help									
Always lay down	5	19.2	1	3.8	0.51	0.083	0.025		
Able to sit up if there is support	5	19.2	2	7.7	0.35				
Able to sit up without support	16	61.5	23	88.5	−0.64				
Condition of motor function: ability to walk around indoors									
Able	15	57.7	22	84.6	−0.61	0.064	–		
Unable	11	42.3	4	15.4	0.61				
Condition of intellectual function									
No response if called	3	13.0	0	0.0	0.74	0.092	0.320		
Turning to the call (voice)	3	13.0	1	4.0	0.34				
Able to follow simple instructions	2	8.7	5	20.0	−0.33				
Able to engage in simple communication	7	30.4	6	24.0	0.14				
Able to participate in daily conversation and group activities	3	13.0	10	40.0	−0.63				
No intellectual dysfunction/delayed	5	21.7	3	12.0	0.26				
SMID									
Yes	9	34.6	1	3.6	0.88	0.004	–		
No	17	65.4	27	96.4	−0.88				
Severe MCDG									
Yes	3	11.5	2	7.1	0.15	0.663	–		
No	23	88.5	26	92.9	−0.15				
Actual utilization of social resources: Medical service									
Yes	7	26.9	4	14.8	0.30	0.327	–		
No	19	73.1	23	85.2	−0.30				
Actual utilization of social resources: Welfare service									
Yes	9	34.6	8	29.6	0.11	0.773	–		
No	17	65.4	19	70.4	−0.11				
Actual utilization of social resources: Daycare service									
Yes	14	53.8	20	74.1	−0.43	0.158	–		
No	12	46.2	7	25.9	0.43				
Actual utilization of social resources: Administration or other services									
Yes	8	30.8	4	15.4	0.37	0.324	–		
No	18	69.2	22	84.6	−0.37				
Age at diagnosis									
Before birth	1	3.8	1	3.7	0.01	0.916	0.719		
0 years old	8	30.8	7	25.9	0.11				
1–3 years old	9	34.6	9	33.3	0.03				
4–6 years old	3	11.5	6	22.2	−0.29				
7–10 years old	3	11.5	3	11.1	0.01				
11–15 years old	2	7.7	1	3.7	0.17				
	*n*	mean	SD	*n*	mean	SD	Effect size (Cohen's *d*)	*P* (Welch *t*-test)	*P* (Mann–Whitney test)
Age at survey	26	9.2	4.5	28	11.6	3.9	−0.59	0.037	0.037
Score of SMID-MCDG	26	4.9	10.2	28	1.5	3.8	0.45	0.119	0.157
	*n*	%		*n*	%		Effect size (Cohen's *h*)	*P* (Fisher exact test)	*P* (Mann–Whitney test)
Outcomes at baseline									
Whether the parent gets up during the night to care for the child									
Every night	7	30.4		2	11.8		0.47	0.329	0.100
Several nights a week	5	21.7		4	23.5				
Several nights a month	6	26.1		3	17.6				
Never	5	21.7		8	47.1				
Do you feel you can properly utilize social resources?									
I never utilize social resources	2	7.7		0	0.0		0.56	0.531	0.721
I do not utilize social resources very often	7	26.9		10	35.7				
I sometimes utilize social resources	11	42.3		10	35.7				
I often utilize social resources	6	23.1		8	28.6				

	*n*	mean	SD	*n*	mean	SD	Effect size (Cohen's *d*)	*P* (Welch *t*-test)	*P* (Mann–Whitney test)
Average hours of sleep per day	26	6.1	1.2	28	5.8	1.5	0.26	0.340	0.446
ZBI-8 (range: 8–40)	26	19.3	6.4	28	19.4	7.6	−0.01	0.972	0.795
SCS-SF									
Total score (range: 12–60)	26	38.2	8.6	28	38.2	9.6	0.01	0.977	0.979
Positive score (range: 6–30)	26	21.1	4.2	28	19.4	5.1	0.36	0.193	0.297
Negative score (range: 6–30)	26	18.8	6.4	28	17.3	5.7	0.26	0.338	0.289
Self-kindness (range: 2–10)	26	7.0	2.1	28	6.5	2.0	0.26	0.337	0.260
Self-judgement (range: 2–10)	26	6.0	2.3	28	5.8	2.2	0.09	0.733	0.760
Common humanity (range: 2–10)	26	6.5	1.8	28	6.1	1.9	0.21	0.449	0.479
Isolation (range: 2–10)	26	6.2	2.7	28	5.4	2.2	0.29	0.289	0.238
Mindfulness (range: 2–10)	26	7.6	1.7	28	6.9	1.9	0.39	0.155	0.202
Over-identification (range: 2–10)	26	6.7	2.2	28	6.0	2.5	0.28	0.308	0.335
SF8 (standardized to mean of 50 and SD of 10)									
PCS	25	62.0	9.4	28	62.5	6.2	−0.06	0.826	0.612
MCS	25	44.3	9.2	28	44.2	8.9	0.00	0.989	0.824
FES									
Total score (range: 34–170)	26	113.0	20.7	28	110.4	25.5	0.11	0.681	0.516
FA (range: 12–60)	26	41.0	7.7	28	40.1	9.3	0.09	0.727	0.467
SS (range: 12–60)	26	42.8	7.1	28	41.4	9.2	0.17	0.540	0.527
SP (range: 10–50)	26	29.2	8.1	28	28.8	8.2	0.05	0.854	0.735

Missing data were excluded.

Cohen's *d*: >0.20 is defined as small, >0.50 is medium, and >0.80 is large difference between means.

Cohen's *h*: >0.20 is defined as small, >0.50 is medium, and >0.80 is large difference between proportions.

FES, family empowerment scale; FA, family (internal) relationships: “What can be done within the family?”; SS, relationships with service systems: “What can be done with service providers?”; SP, involvement with community: “What can be done with government officials in the local community?; ZBI-8, Zarit Caregiver Burden Interview; SCS-SF, the short form of the self-compassion scale; SF8, the MOS Short Form 8-Item Healthy Survey; PCS, physical component summary; MCS, mental component summary; SMID, severe motor and intellectual disabilities; defined as the disability to walk around indoors and perform simple communication; Severe MCDG, medical care-dependent group; group comprises children with SMID-MCDG score of 10 or more.

About 70% of the children were boys between the ages of 2–20 years. The waitlist-controlled group was significantly older. The age at diagnosis ranged from prenatal to 15 years, with diagnoses including chromosomal abnormality, autism spectrum disorder, Down's syndrome, psychomotor retardation, intellectual disabilities, higher-order brain dysfunction, epilepsy, and cerebral palsy, among others. The intervention group had many children with motor function difficulties and a significant majority had severe motor and intellectual disabilities. There was no difference in the score of “Severe Motor and Intellectual Disabilities-severe Medical Care Dependent Group” (indicating the degree of required medical care). CWD of parents in both groups received similar levels of varied medical and non-medical care (ventilator and other respiratory care, enema/stool extraction, changing position, etc.) (Table 2).

### Outcome comparison between groups

**Figure 2:**
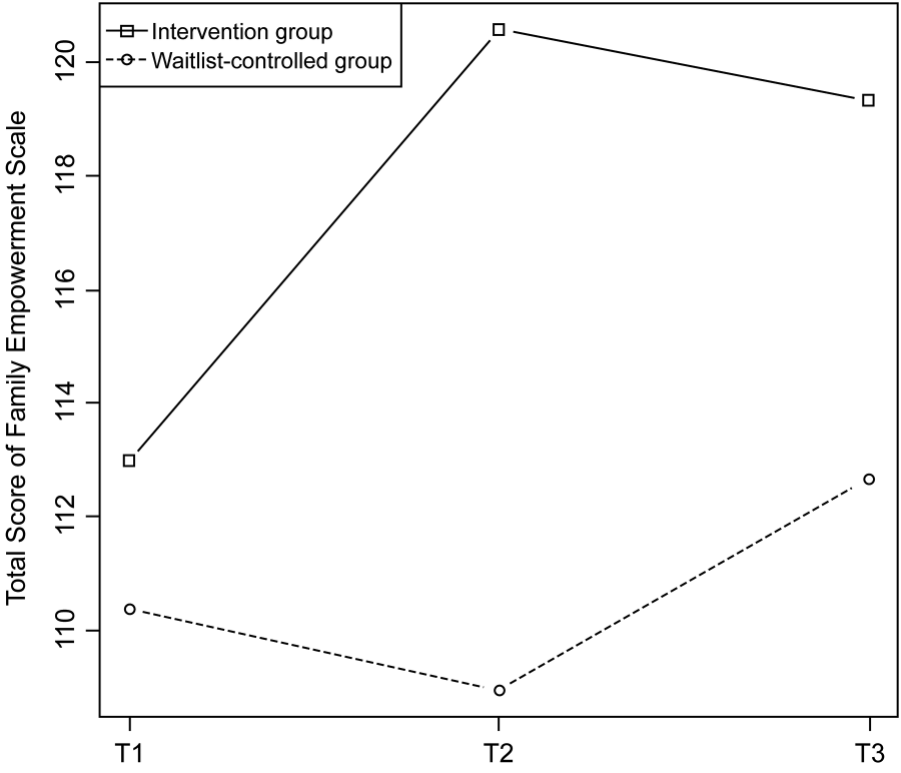
Changes in the total score of the family empowerment scale.

**Figure 3:**
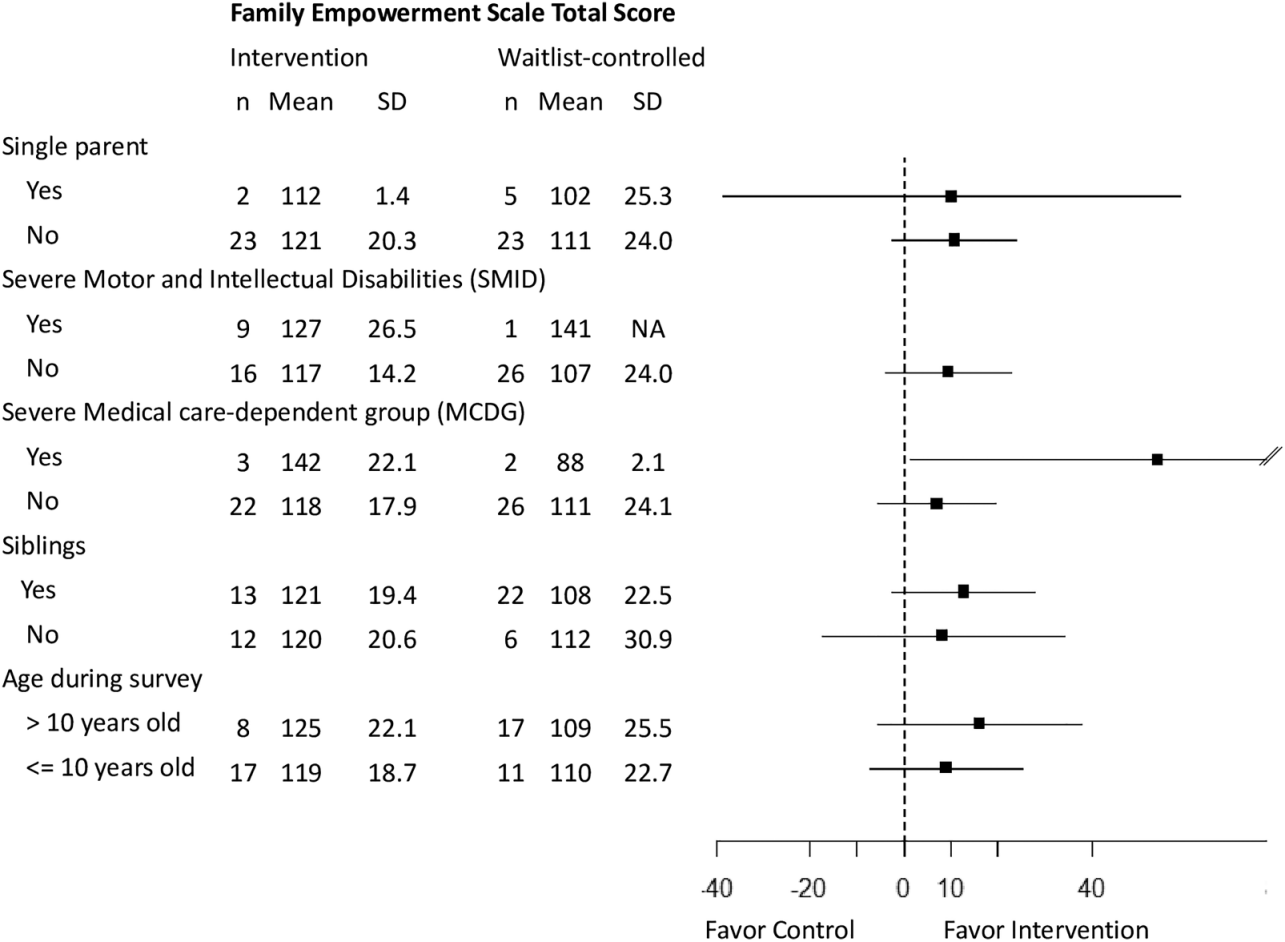
Subgroup analysis.

The outcome scores at T1 were not significantly different between the groups (Table 2). Figure 2 shows the changes in the FES total score; after adjusting for the value at T1, the score was significantly higher at T2 in the intervention group than in the waitlist-controlled group, with a medium effect size (Table 3). The same trend was observed in the sensitivity analysis and became stronger in the per-protocol analysis. Family empowerment of participants among the intervention group surely increased after the intervention. Subgroup analyses were performed on the pre-planned subgroups and background attributes that differed between the groups at T1 (Figure 3). The results were consistent across most subgroups, with the intervention group tending to score higher than the control group. The increases in family empowerment were observed overall (not among specific subgroups).

**Table 3:** Outcomes of the early intervention group and the waitlist-controlled group.

	Intervention group			Waitlist-controlled group			Effect size (Cohen's *d*)	Test type	*P*	Effect size
	*n*	Mean	SD	*n*	Mean	SD				
Primary outcomes										
FES total score (range: 34–170) at T2	25	120.6	19.59	28	109.0	23.97	0.53	ANCOVA^a^	0.003	0.176
Sensitivity analysis for the primary result (FES total score at T2)										
Per-protocol analysis	20	124.0	19.71	20	106.1	22.71	0.84	ANCOVA^a^	0.000	0.382
Adjustment for baseline imbalance	25	120.6	19.59	27	108.6	24.36	0.54	ANCOVA^b^	0.018	0.191
Consideration of dropout (regarded as no change)	26	120.2	19.29	28	109.0	23.97	0.51	ANCOVA^a^	0.003	0.166
	*n*	%		*n*	%		Effect size (Cohen's *h*)			
Secondary outcomes at T2										
Whether the parent gets up during the night to care for the child										
Every night	6	30.0		1	5.3		0.70	MWU^d^	0.034	−0.384
Several nights a week	3	15.0		4	21.1		−0.16			
Several nights a month	7	35.0		4	21.1		0.31			
Never	4	20.0		10	52.6		−0.70			
Do you feel you can properly utilize social resources?										
I never utilize social resources	8	32.0		7	25.9		0.13	MWU^c^	0.407	−0.123
I do not utilize social resources very often	14	56.0		14	51.9		0.08			
I sometimes utilize social resources	2	8.0		4	14.8		−0.22			
I often utilize social resources	1	4.0		2	7.4		−0.15			
	*n*	Mean	SD	*n*	mean	SD	Effect size (Cohen's *d*)			
Average hours of sleep per day	26	6.2	1.21	28	5.7	1.39	0.32	ANCOVA^a^	0.471	−0.005
ZBI-8 (range: 8–40)	25	18.8	6.14	28	20.9	8.18	−0.29	ANCOVA^a^	0.031	0.082
SCS-SF										
Total score (range: 12–60)	25	41.5	9.16	28	37.6	9.62	0.41	ANCOVA^a^	0.038	0.079
Positive score (range: 6–30)	25	22.8	4.12	28	20.5	5.13	0.48	ANCOVA^a^	0.214	0.026
Negative score (range: 6–30)	25	17.3	5.82	28	18.9	5.84	−0.28	ANCOVA^a^	0.004	0.167
Self-kindness (range: 2–10)	25	7.6	1.76	28	6.6	2.17	0.50	ANCOVA^a^	0.135	0.040
Self-judgement (range: 2–10)	25	5.8	2.31	28	6.1	2.34	−0.15	ANCOVA^a^	0.199	0.022
Common humanity (range: 2–10)	25	7.4	1.96	28	6.7	2.00	0.36	ANCOVA^a^	0.259	0.023
Isolation (range: 2–10)	25	5.4	2.33	28	5.7	2.27	−0.15	ANCOVA^a^	0.023	0.095
Mindfulness (range: 2–10)	25	7.8	1.58	28	7.3	1.96	0.29	ANCOVA^a^	0.603	0.002
Over-identification (range: 2–10)	25	6.1	2.40	28	7.0	2.30	−0.39	ANCOVA^a^	0.003	0.178
SF8 (standardized to mean of 50 and SD of 10)										
PCS	24	61.0	9.18	28	60.6	7.82	0.05	ANCOVA^a^	0.571	−0.003
MCS	24	46.2	8.04	28	43.4	8.31	0.35	ANCOVA^a^	0.055	0.066
FES										
FA (range: 12–60)	25	43.6	7.11	28	39.5	8.84	0.50	ANCOVA^a^	0.009	0.128
SS (range: 12–60)	25	45.9	7.11	28	41.0	8.47	0.62	ANCOVA^a^	0.003	0.183
SP (range: 10–50)	25	31.1	6.90	28	28.4	8.49	0.35	ANOVA^d^	0.208	0.175

Missing data were excluded.

FES, family empowerment scale; FA, family (internal) relationships: “What can be done within the family?”; SS, relationships with service systems: “What can be done with service providers?”; SP, involvement with community: “What can be done within the local community?”; ZBI-8, Zarit Caregiver Burden Interview; SCS-SF, the short form of the self-compassion scale; SF8, the MOS Short Form 8-Item Healthy Survey; PCS, physical component summary; MCS, mental component summary; SMID, severe motor and intellectual disabilities; defined as the disability to walk around indoors and perform simple communication; severe MCDG, medical care-dependent group; group comprises children with SMID-MCDG score of 10 or more.

^a^ ANCOVA with baseline (T1) score adjustment. Effect size f2: small effect = 0.02, medium effect = 0.15, large effect = 0.35 (32).

^b^ ANCOVA like “a”, but adjustment also with imbalance variables at baseline (T1); living with siblings, SMID, age of children at survey.

^c^ MWU, Mann–Whitney *U* test. Effect size delta: small effect = 0.2, medium effect = 0.5, large effect = 0.8 (33).

^d^ ANOVA because there was a significant interaction between the group and the baseline (T1) score. Effect size f: small effect = 0.10, medium effect = 0.25, large effect = 0.40 (32).

Regarding the secondary outcomes, the FES subscale scores for FA and SS were significantly higher in the intervention group (Table 3). Our program empowered participants, especially for family coordination and social service utilization (compared to community/political advocation). The ZBI-8 score for caregiver burden was lower in the intervention group, the mean sleep time was not different, and those in the intervention group woke up more frequently for nighttime care. Our program worked as hypothesized; it decreased caregiver burden and so family empowerment was increased. And our program did not impact on shortening sleep time. There was no between-group difference in whether participants felt they could properly utilize (be aware of) social resources. Regarding improvement in social resource utilization after the program, 13 participants in the T2 intervention group responded with “Can utilize it more” and 10 reported it to be “Unchanged”; however, none of them said they “No longer utilize it.” In the waitlist-controlled T3 group, six reported that they “Can utilize it more” and 16 responded with “Unchanged,” but none of the participants said they “No longer utilize it.” Participants of the intervention had no increased utilization of services, in fact, however, they surely felt an improvement in utilization of services. Regarding self-compassion, the intervention group had a higher total score, a lower negative score, and lower scores on isolation and over-identification. Our program worked as hypothesized; it increased self-compassion and so family empowerment was increased. Further, the increase of self-compassion can be focused more on resolving negative aspects than on enforcing positive aspects of self-compassion. There was no difference in scores between the groups in health-related QOL (SF-8 score).

### Sustainability and reproducibility of main outcome

The FES (total, FA, SS) and SCS-SF (total, common humanity, isolation) scores were the outcomes that changed significantly from T1 to T2 in the intervention group. Except for the common humanity subdomain, changes in all the outcomes were sustained until T3 in the intervention group. Furthermore, FES SP and SCS negative scores and over-identification changed significantly from T1 to T3 (Table 4). The effects of our intervention observed at T2 were sustained to T3.

**Table 4:** Maintenance of the intervention effects in the early intervention group and reproducibility of the effect in the delayed (waitlist) group.

	T1 → T2 (Intervention group)					T1 → T3 (Intervention group)					T2 → T3 (Waitlist-controlled group)				
*n*	Mean change	SD	*P*	Effect size (Glass's Δ)	*n*	Mean change	SD	*P*	Effect size (Glass's Δ)	*n*	Mean change	SD	*P*	Effect size (Glass's Δ)
FES	Total score	25	7.5	14.06	0.014	0.35	23	7.9	10.03	0.001	0.37	22	6.2	13.27	0.039	0.25
	FA	25	2.4	5.23	0.029	0.31	23	1.8	3.06	0.009	0.23	22	2.6	4.82	0.018	0.28
	SS	25	3.0	5.28	0.009	0.41	23	2.8	4.78	0.011	0.38	22	2.1	5.10	0.068	0.23
	SP	25	2.0	5.61	0.081	0.25	23	3.3	4.56	0.002	0.39	22	1.5	5.05	0.178	0.18
Average hours of sleep per day		26	0.0	0.71	0.783	0.03	23	0.1	0.81	0.610	0.08	22	0.2	0.48	0.059	0.21
ZBI-8		25	−0.8	3.50	0.268	−0.12	23	0.0	5.85	1.000	0.00	22	−0.8	5.16	0.490	−0.09
SCS-SF	Total score	25	3.3	7.71	0.041	0.38	23	3.9	6.92	0.014	0.43	23	2.1	4.86	0.047	0.23
	Positive score	25	1.7	4.85	0.096	0.39	23	1.3	4.25	0.155	0.29	23	1.3	2.62	0.026	0.24
	Negative score	25	−1.6	4.40	0.074	−0.25	23	−2.6	4.04	0.006	−0.40	23	−0.8	3.28	0.241	−0.15
	Self-kindness	25	0.5	2.06	0.220	0.25	23	0.6	2.15	0.188	0.29	23	0.5	1.24	0.077	0.21
	Self-judgment	25	−0.2	1.83	0.519	−0.10	23	−0.7	2.04	0.139	−0.27	23	−0.2	1.19	0.492	−0.08
	Common humanity	25	1.0	2.24	0.043	0.51	23	0.6	2.06	0.202	0.30	23	0.5	1.34	0.102	0.23
	Isolation	25	−0.8	1.71	0.028	−0.29	23	−1.0	1.46	0.002	−0.39	23	0.0	2.10	0.922	−0.02
	Mindfulness	25	0.2	2.02	0.625	0.11	23	0.1	1.36	0.650	0.08	23	0.3	1.11	0.148	0.19
	Over-identification	25	−0.6	1.85	0.118	−0.27	23	−0.9	1.52	0.012	−0.38	23	−0.6	1.44	0.055	−0.27
SF8	PCS	24	−0.7	5.70	0.558	−0.07	22	−0.1	9.16	0.948	−0.01	22	−0.3	5.39	0.778	−0.04
	MCS	24	2.3	7.75	0.156	0.25	22	3.2	8.47	0.091	0.33	22	1.5	6.63	0.286	0.18

Missing data were excluded.

Glass's Δ, the mean change divided by the standard deviation of the former (baseline) time point;FES, family empowerment scale; FA, family (internal) relationships: “What can be done within the family?”; SS, relationships with service systems: “What can be done with service providers?”; SP, involvement with community: “What can be done within the local community?”; ZBI-8, Zarit Caregiver Burden Interview; SCS-SF, the short form of the self-compassion scale; SF8, the MOS Short Form 8-Item Healthy Survey; PCS, physical component summary; MCS, mental component summary.

Of those outcomes, the significant changes in the FES total, FA, and SCS total scores could be reproduced from T2 to T3 in the waitlist-controlled group (Table 4).

### Process evaluation

Most participants rated the program positively. The intervention group members who responded with “not very good” at T3 stated that they were already experiencing emotional distress and that they found attending the weekly sessions exhausting, causing them to feel depressed. More than 80% of the participants told their families about the program's content—most during the program (until T2) and some during the first month after the program ended (between T2 and T3). Most said they would recommend the program to their friends. Common reasons for not recommending were lack of friends; difficulty in using the online system, Zoom; the belief that people would not be interested in family empowerment; and the prior existence of a support system. While many participants suggested making the program available to people who want it, some suggested establishing a system that everyone could participate in, as an effective way of reaching those unable to obtain information on their own.

Some participants reported wanting to attend before their child started school; others said people should be able to attend based on their preference. Several participants suggested that groups should have wide age ranges. None of the participants felt that the number of sessions should be reduced (Supplementary Table S1).

## Discussion

### Subject characteristics

Most participants in our study were mothers. In many families, mothers are the primary caregivers for CWD. In cases of needing much medical care with severe disabilities, mothers commit their time mainly to childcare and housework and do not hold formal jobs (34–36), a reality reflected in this study. Many participants also had a university degree or higher. This suggests the possibility that people with a certain level of knowledge and education were attracted to a program promoting “family empowerment,” not a familiar term in Japanese culture. Other family participation-related programs have also attracted highly educated people (37). Nevertheless, this program is intended for all caregivers, regardless of their educational background. We needed to devise ways of appealing to a wide range of the population using simple language.

It is notable that our program had few dropouts. Mechanisms to prevent participant dropout during programs are needed. Measures to encourage participation included aligning program goals with participants' motivations, facilitating accessibility by offering flexible hours and locations, and providing opportunities for exchanging opinions in a group (38). In another intervention study, participants stayed connected to their identified goals during sessions and could direct their resources to achieve them; they reported that the time and mechanism for understanding everything by themselves was beneficial (39). In this study, participants were allowed to ask for a summary of the program before applying for inclusion. The program was held multiple times, giving them the flexibility to apply each month and participate online from home. To sustain their sense of involvement, homework was included, which was later discussed in small group meetings. Our low dropout rate can be attributed to these participation drivers.

### Effects of the program intervention

After the program, the intervention group's FES FA and SS scores increased, possibly due to participants' improved understanding of how they can collaborate with their families, with service providers, and practice problem-solving techniques. This was our intention behind designing this family empowerment program. Strategies for boosting access to information, counseling, and community support services are likely to empower families and optimize their health and well-being (40). These findings are consistent with our program's strategy.

However, the FES SP score did not increase significantly. SP is composed of the initials Social/Political. It is one of the three aspects of FES, meaning “involvement with the community”.

Previous studies confirmed floor effects in several SP items, especially in Japanese suburbs (26). Unlike other levels of family empowerment, it was assumed that accessing new targets (administrative officials and politicians) would be difficult, and even if that happened, it would take some time to build a cooperative system. Thus, SP needs to be examined over a longer period for evaluating its effectiveness.

In our study, the intervention group's care burden lessened. Studies have reported a negative correlation between parental stress and family empowerment (41). Caregivers can be empowered through informal resources, such as family, friends, and support groups, to reduce the care burden (4). Although the childcare needs did not change, sharing their worries and problems through this program and knowing that they had people to talk to about their challenges enhanced participants' awareness of daily care, thereby easing their care burden. This provides supporting evidence for the FEM, which aims to increase family empowerment by reducing the care burden (14).

Conversely, there was no effect on the awareness of social resource utilization, possibly because our focus was on participants' awareness of their use of services rather than the types of services being used. It is difficult for caregiver families to search for and find appropriate social resources to meet their requirements and use social welfare systems (42, 43). The study sample learned about new services through the program but faced several barriers in utilizing them, and thus did not reach the utilization stage. However, the awareness and use of social resources are important variables associated with family empowerment and easing care burden (14), and should be used as indicators for program assessment. The actual use of and need for social resources should be examined separately from the participants' ability to utilize them. The utilization and awareness of using social resources need to be studied more comprehensively, possibly by developing appropriate measurement scales.

We found that the self-compassion of participants improved, especially in the isolation and over-identification domains. Caring for a child with a disability creates physical, psychological, and social stress as well as feelings of self-reproach and low self-efficacy (3, 44). Caregivers lose opportunities for social participation, such as employment, and this worsens their mental health (5, 6). Participating in the group-based program was a form of social participation that seemed to help reduce their loneliness. In the program, participants exchanged opinions with others with different attributes (age of children, degree of disabilities or medical dependence, area of residence, etc.), allowing them to realize how to find a balance between joy and stress.

The effects on FES FA and SS and isolation persisted even after the program ended. While the impact of intervention programs is limited to the intervention period (18), this study's finding is important in evaluating the program's ripple effects. When a person is empowered, even if their situation or that of their family changes, they can continue to care for their child while dealing with such changes (7). This program, when coupled with the characteristics of family empowerment, can be expected to have significant ripple effects in society.

### Managing and promoting the program

We demonstrated that a program developed using the evidence-based methods of the IM approach and BCTs, based on theoretical foundations of previous studies, can improve family empowerment. Due to COVID-19, medical and welfare services that support CWD have been shifted online (45, 46), and studies on online educational programs' effectiveness are being conducted (47). Digital tools to enhance family empowerment have been developed in the Netherlands and their effects have been confirmed (48). Parents of children and youth with disabilities reported spending many hours searching the Internet for resources and learning new skills (such as nursing care and therapies) while navigating the system, advocating for their children, fundraising, and providing care (49). In this context, our online program allowed people to apply regardless of region, location, or the child's disability. Interacting with participants in various situations allowed them to evaluate their own situations from new perspectives and discuss the SS available in different regions. This may help expand their care network in the future.

For caregivers of CWD, using the online platform eliminated the barrier of traveling to another location. We could take advantage of the benefits of online meetings to demonstrate the program's effectiveness.

### Limitations and future research

As participation was voluntary, only caregivers with an active interest in participating in online programs may have been included. However, this program was intended for all caregivers. The generalizability of the program's effectiveness could be enhanced by including people who are not Internet savvy, are reluctant to participate in such programs, or lack the resources to participate.

The questionnaires were self-administered. Although it provided subjective assessments, changes in participants' situations also need to be understood objectively. In future studies, we intend to conduct third-party surveys through other family members and service providers to obtain a multifaceted assessment of our program.

The subgroup analyses had small sample sizes that were insufficient for examining associations among multiple attributes and their impact on the program's effects. While continuing its implementation, further investigation of the effects of people's attributes on their participation in the program is needed.

### Conclusion

In this study, using the IM approach and BCTs, we deployed an online peer support program to promote behavioral change and empower families raising CWD all over Japan, and tested its effectiveness.

After the completion of the program, participants' family empowerment increased, especially for family coordination and service utilization. Backgrounds of the increase were self-compassion improvement and easing their care burden. The program's effect on family empowerment and self-compassion was sustained even after 1 month of the program. Results demonstrated the program's effectiveness in promoting family empowerment; hence, full-scale social implementation of the program in the future can be expected.

However, the program had no effect on recognizing the use of social resources, which is an important element that contributes to care burden reduction and family empowerment. Thus, the program content should be examined with a focus on this element.

Our online program may have promoted the ease of participation for caregivers of CWD who find traveling difficult, by allowing them to attend the program regardless of region or type of disability and facilitated the expansion of their care network. However, the challenge ahead lies in achieving full-scale social implementation of this program, accumulating and generalizing the effects, and performing a multifaceted evaluation of the program and its effects.

## Data Availability

The original contributions presented in the study are included in the article/Supplementary Material, further inquiries can be directed to the corresponding authors.
