# Supplementary material for: Effectiveness of a peer group-based online intervention program in empowering families of children with disabilities at home
Source: Front Pediatr. 2022 Oct 24;10:929146. doi: 10.3389/fped.2022.929146 (PMC9638189; doi:10.3389/fped.2022.929146)
Supplement: Supplementary file 1 [file Datasheet1.pdf]

Supplementary Material

Supplementary Figure 1. The SPIRIT schedule of enrolment, interventions, and assessments

	STUDY PERIOD			
	Enrolment	Allocation	Post-allocation	
Timepoint(T)	T_1	0	*Post-intervention* T_2	*Four weeks post-intervention* T_3
**ENROLMENT:**				
Eligibility screen	X			
Informed consent	X			
Allocation		X		
**INTERVENTIONS:**				
Intervention/early group		◆———	———◆	
Waitlist-controlled/delayed group			◆———	———◆
**ASSESSMENTS:**				
Demographics of participants and their children and families	X		X	X
Short version of the Zarit Caregiver Burden Interview (J-ZBI_8; 8 items)	X		X	X
Awareness of social resource utilization	X		X	X
Short form of the Japanese version of the Self-Compassion Scale (SCS-12; 12 items)	X		X	X
QOL of primary caregiver (SF-8; 8 items)	X		X	X
Japanese version of the Family Empowerment Scale (J-FES; 34 items)	X		X	X
Feedback of program			X	X

	(Early intervention group only)

⟷ **: Intervention period, X: At the time of collection**

Supplementary Table 1. Direct opinions of participants about the family empowerment program after participation

	Total		Intervention group				Waitlist-controlled group	
			T2		T3		T3	
	n	%	n	%	n	%	n	%
How did you feel after attending the program?								
Very good	46	69.7	17	73.9	14	70.0	15	65.2
Good	13	19.7	4	17.4	4	20.0	5	21.7
Somewhat good	6	9.1	2	8.7	1	5.0	3	13.0
Not very good	1	1.5	0	0.0	1	5.0	0	0.0
Not good	0	0.0	0	0.0	0	0.0	0	0.0
Not good at all	0	0.0	0	0.0	0	0.0	0	0.0
Did you tell your family about the content of the program and the information you learned?								
I did	30	44.8	10	41.7	12	57.1	8	36.4
A little	26	38.8	10	41.7	7	33.3	9	40.9
Not much	4	6.0	1	4.2	0	0.0	3	13.6
Not at all	7	10.4	3	12.5	2	9.5	2	9.1
Would you recommend the program to your friends?								
Yes	48	75.0	19	82.6	15	78.9	14	63.6
Probably	13	20.3	4	17.4	1	5.3	8	36.4
Not really	2	3.1	0	0.0	2	10.5	0	0.0
No	1	1.6	0	0.0	1	5.3	0	0.0
How could the program be expanded?								
People who want to take it should do so	49	69.0	15	57.7	15	68.2	19	82.6
Everyone should take it *	19	26.8	9	34.6	6	27.3	4	17.4
There's no need	3	4.2	2	7.7	1	4.5	0	0.0
Opinion about the number of sessions								
Fewer is better	0	0.0	0	0.0	0	0.0	0	0.0
This amount is good	51	76.1	16	69.6	20	95.2	15	65.2
More is better	16	23.9	7	30.4	1	4.8	8	34.8

Missing data were excluded.

*; To be precise, "It is basically good for everyone who has child with a disability to take this course, similar to what is done with heath checks."
